# Genetic Characterization of Hydatid Cysts Isolated from Domestic Animals in Lorestan Province, Western Iran

**Published:** 2018

**Authors:** Farnaz KHEIRANDISH, Ebrahim BADPARVA, Hossein MAHMMOUDVAND, Elahe BEIRANVAND, Simin BABAEI, Bahram NASIRI

**Affiliations:** 1. Razi Herbal Medicines Research Center, Lorestan University of Medical Sciences, Khorramabad, Iran; 2. Social Determinants of Health Research Center, Lorestan University of Medical Sciences, Khorramabad, Iran; 3. Dept. of Medical Parasitology and Mycology, School of Medicine, Jundishapur University of Medical Sciences, Ahvaz, Iran; 4. Student Research Committee, Lorestan University of Medical Sciences, Khorramabad, Iran

**Keywords:** Cystic echinococcosis, Cox1, Sheep, *Echinococcus granulosus*

## Abstract

**Background:**

Regarding hydatid cyst (cystic echinococcosis, CE) as a human public health problem in the West of Iran, molecular data related to the genotypes of *Echinococcus granulosus* in cattle and sheep in these regions are still insufficient. Here, we evaluated the genotypes of *E. granulosus* infecting sheep and cattle in western Iran.

**Methods:**

Totally, 36 hydatid cysts including 18 hydatid cysts of sheep and 18 hydatid cysts of cattle were collected from Khorramabad slaughterhouse (Lorestan Province), Western Iran between May to September 2014. Protoscoleces or germinal layers were collected from individual cysts, DNA was extracted, and genotyping was performed by sequencing and analyzing mitochondrial cytochrome *c* oxidase subunit 1 (*cox*1) gene.

**Results:**

In sequencing analysis, all of sheep isolates belonged to genotype G1 (sheep strain). Among cattle hydatid cyst isolates, 16/18 (88.9%) were belonged to genotype G1 and 2/18 (11.1%) were belonged to G3 genotype. The phylogenetic analysis showed two clusters; one of the clusters includes cattle G3 genotype and the other cluster represents sheep and cattle G1 genotype that were isolated.

**Conclusion:**

The common sheep strain/G1 is predominant genotype in the western part of Iran, followed by G3 genotype, circulating among the animal hosts in this region. Further studies covering a larger number of isolates might be necessary to see if there are other genotypes in the hydatid cyst population in this region of Iran.

## Introduction

Hydatid cyst (cystic echinococcosis, CE), a parasitic infection caused by the larval stage of dog tapeworm *Echinococcus granulosus*, has been identified as a major public health and economic problem in developing countries, including Iran (1). The dog is the definitive host, where adult tapeworms attached to the intestinal epithelium, and humans as well as domestic livestock can be the intermediate host, where the worms form hydatid cysts in various organs (2).

In CE, clinical manifestations are mostly related to the localization, size, and number of cysts. There seemed to be a relationship between the genotypes and the size of hydatid cysts, in which all the patients infected with G7 genotype showed smaller liver cysts than those infected with G1 genotype (3). Molecular epidemiological data have identified 10 distinct genotypes of *E. granulosus* (G1–G10): *E. granulosus sensustricto* (G1, G2, G3), *Echinococcus equines* (G4), *E. ortleppi* (G5), *E. intermedius* (G6, G7, G8, G10) (4). In Iran, to date, three genotypes have been found including G1, G3, and G6 genotypes in humans and animals in different regions (5). Sheep, buffalo, and camels that are among common livestock in Iran are considered as main reservoirs of genotypes G1, G3, and G6, respectively (6). Reviews on the incidence of *E. granulosus* have reported that G1 (common sheep strain) is the most predominant genotype in Iran (6) and also most parts of the world (4). Studies have demonstrated that the, cytochrome *c* oxidase subunit 1 (*cox*1) and NADH dehydrogenase 1 (*nad*1) genes are good molecular markers for investigating genetic variation in a number of isolates of *E. granulosus* from a range of hosts (i.e., cattle, sheep, and goat) in Iran (5, 7). Moreover, mitochondrial genomes have the ability to eliminate some limitations in *Echinococcus* taxonomy (8).

Recently, Parsa et al (9) have demonstrated three genotypes (G1 [75%], G2 [10%] and G3 [15%]) from the *E. granulosus*isolates of 71 stray dogs, from Lorestan province, western Iran using DNA sequencing of the partial mitochondrial *cox1* and *nad1* (9). However, no genotyping data are available about *E. granulosus* isolates of domestic livestock in this province using DNA sequencing of *cox1* and *nad1*.

Therefore, the present study aimed molecularly identify and genotype hydatid cysts of some livestock (sheep, and cattle) in Lorestan Province by sequencing and analyzing mitochondrial *cox1* gene, to speculate on possible transmission patterns of this cestode by homology analysis, and to understand the phylogeny of genotypes of *E. granulosus* by constructing neighbor-joining trees.

## Materials and Methods

### Ethics statement

This study was approved by the Committee on the Ethics of Animal and Human Experiments of the Lorestan University of Medical Science, Khorramabad, Iran (Permit Number: 89/6).

### Collection of hydatid cyst samples

A total of 36 hydatid cysts including 18 hydatid cysts of sheep and 18 hydatid cysts of cattle were collected from Khorramabad slaughterhouse (Lorestan Province), Western Iran between May to September 2014 and carried to the Parasitology Laboratory at the, Lorestan University of Medical Sciences, (Khorramabad, Iran).

### Microscopic examination of protoscoleces

To confirm whether or not the protoscoleces exist in hydatid cysts, their contents were subjected to the microscopic examination. Protoscoleces and/or germinal layers were collected from cysts and washed five times with phosphate-buffered saline (PBS). The obtained pellets were fixed in 70 % ethanol and stored at 4 °C until genomic DNA isolation.

### Extraction of genome DNA

To extract the genomic DNA (gDNA) of hydatid cyst samples, protoscoleces and/or germinal layers from each individual cyst were washed five times in sterile-distilled water by centrifugation to remove the ethanol and gDNA of each sample was extracted by High Pure PCR Template preparation kit (Roche, Mannheim, Germany) according to the manufacturer’s instructions. To ensure the accuracy of DNA extraction, concentration of the extracted DNA samples were analyzed by NanoDrop. The extracted DNAs were stored at −20^°^C until required for PCR.

### Mitochondrial PCR amplification

The mitochondrial *cox1* gene was amplified using specific primer sets JB3/JB4.5 (10). PCR was performed in a 25 μl final volume containing 1 X PCR buffer, 2.5 mM MgCl_2_, 0.2 mM of each deoxynucleotide triphosphate (dNTP), 0.2 μM of each primer and 1.5 μM Ampli-Taq Polymerase. Two primers, JB3 (forward), 5′-TTT TTT GGG CAT CCT GAG GTT TAT-3′ and JB4.5 (reverse), 5′-TAA AGA AAG AAC ATA ATG AAA ATG-3′ (10), were used to amplify a 450 bp fragment of *cox1* gene under the following conditions: initial denaturation 94°C for 5 min, followed by 30 cycles of denaturation at 94°C for 30s, annealing at 55°C for 30s and elongation for 45s at 72°C. Final extension was performed at 72°C for 10 min, and then the product of PCR was electrophoresed on 1.5% agar gel. All PCR amplifications were run with negative control (non-template water control).

### Sequencing analysis

All PCR products were subjected to automated sequencing by the Illumina Genome Analysis System, employing the same primers used in the primary PCR. The electropherogram of each sequence was checked by eye, and the sequences were compared with each other using the software BioEdit. Accuracy of the sequencing data was confirmed by sequencing in both directions. Nucleotide sequences obtained in the present study were subjected to BLAST searches (http://www.ncbi.nlm.nih.gov/blast/), and were then aligned and analyzed with each other and *E. granulosus* reference sequences downloaded from GenBank using Clustal X 1.83.

### Phylogenetic analysis

**Fig. 1:**
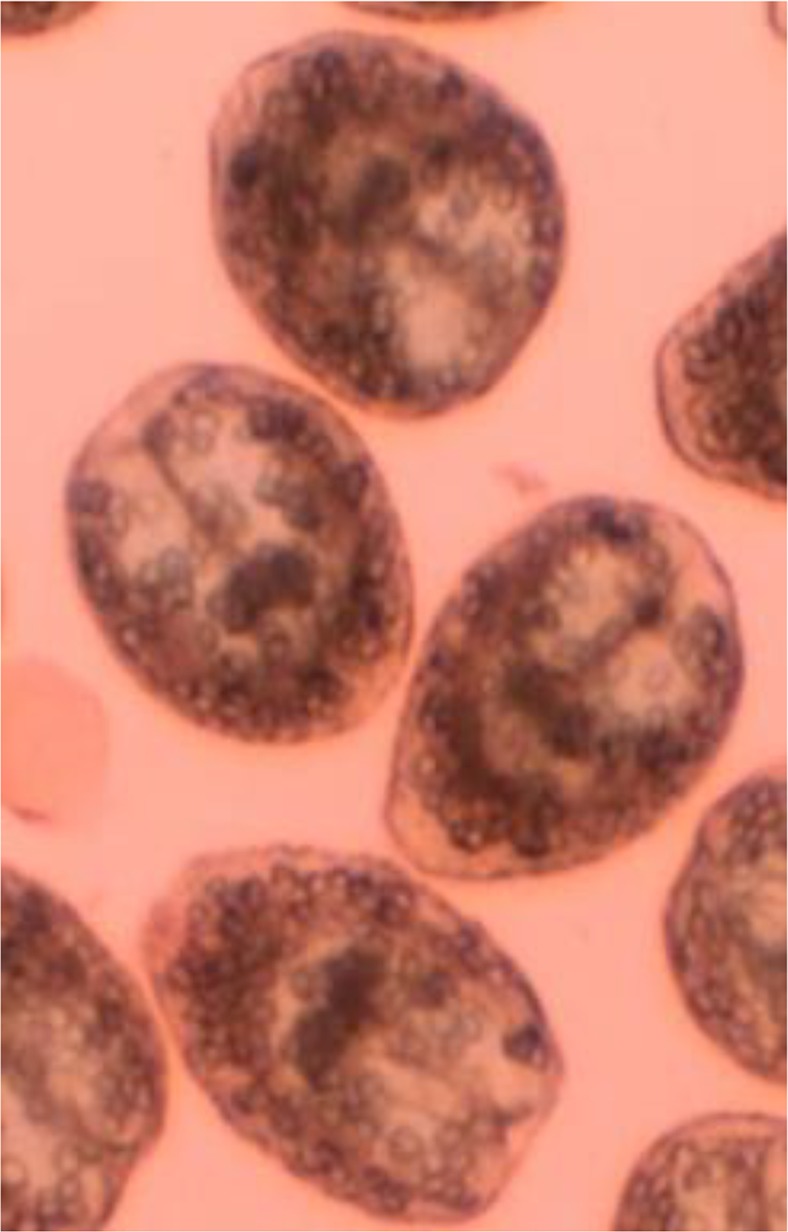
Live (colorless) protoscoleces of hydatid cysts with 0.1% eosin

Sequences were aligned by means of the CLUSTALW software package (www.ebi.ac.uk/clustalw) and analyzed using the Neighbor–Joining (NJ) method provided in the MUST software package. The phylogenetic tree was run using sequences obtained in this study as well as reference sequences available for *E. granulosus* G1(accession number, KT200223.1) and G3 (accession number, HM563022.1) genotypes as shown in Fig. 1. Some of G1 (DQ062857.1, JX878690.1) and G3 (KT074949.1, JN604105.1, KF731907, JX854031.1) genotypes in the gene bank was used for comparison. *E. multilocularis* COX1 (KT318128.1) was applied in the model as anout-group.

## Results

Totally, 36 hydatid cysts isolates from domestic animals from Lorestan Province were tested for the molecular analysis. All these samples were analyzed using mitochondrial *cox1* primers. For all of isolates, fragment of about 450 bp were successfully PCR-amplified within *cox1* gene. In sequencing analysis, the alignments of the sequences determined in this study with those of know genotypes of *E. granulosus* demonstrated all of sheep isolates belonged to genotype G1 (sheep strain). Among cattle hydatid cyst isolates, 16/18 (88.9%) were belonged to genotype G1 and 2/18 (11.1%) were belonged to G3 genotype. All the hydatid cysts belonged to G1 were confirmed to be fertile (with protoscoleces) by microscopy (Fig.1); whereas both hydatid cysts of belonged to G3 were observed to be fertile. Representative nucleotide sequences obtained in this study were deposited in the GenBank database under the accession numbers LC068914.1 and LC068958.1 for G1 genotype (for both sheep and cattle) and G3 genotype (cattle) respectively.

**Fig. 2:**
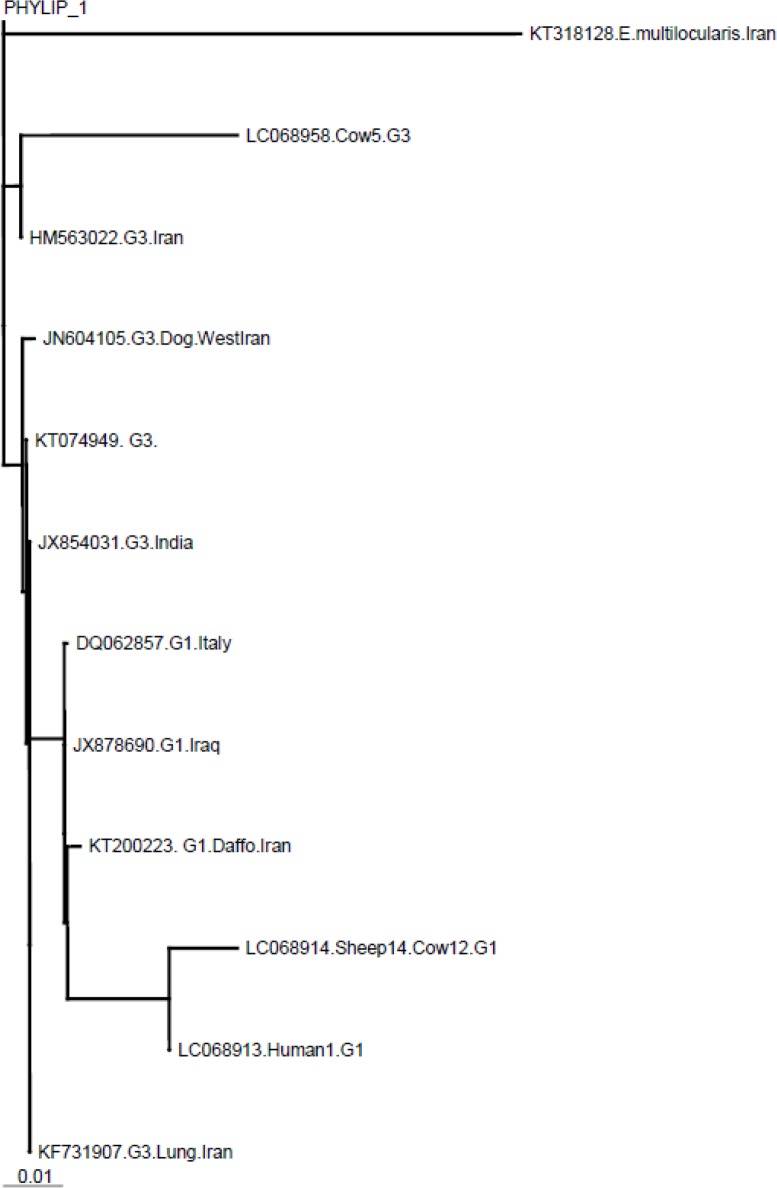
Molecular phylogenetic tree of 3 *E. granulosus* isolates of sheep, cattle, and human along with reference isolates based on CO1 gene sequence. Accession numbers of KT200223 and HM563022 represent reference sequences of *E. granulosus* genotypes G1 and G3, respectively. Some of G1 (DQ062857, JX878690) and G3 (KT074949, JN604105, KF731907, JX854031) genotypes was used for comparison. *E. multilocularis* KT318128 was used as out group sequence data

The phylogenetic analysis showed two clusters; one of the clusters includes cattle G3 genotype and the other cluster represents sheep and cattle G1 genotype that were isolated during in this study (Fig. 2).

## Discussion

Lorestan Province due to favorable weather conditions and the spread of livestock is one of the prone provinces in Iran for prevalence of parasites (11–16). Cystic echinococcosis is a globally distributed parasitic infection of humans and livestock. At present, the World Health Organization (WHO) included CE in a subgroup of selected Neglected Tropical Diseases (NTDs) to be addressed within its 2008–2015 strategic plans for control of NTDs (1). In the present study, 36 hydatid cysts including 18 hydatid cysts of sheep and 18 hydatid cysts of cattle were tested for the molecular analysis. In this study, 34 (94.4%) and 2 (5.6%) of isolates were identified as G1 and G3 genotype of *E. granulosus*, respectively*.* The results presented here demonstrated that G1 was the dominant genotype of CE in some livestock animals such as sheep and cattle in Western Iran. The obtained findings revealed that all the hydatid cysts of isolated from sheep and 16/18 (88.8%) of cattle isolates were belonged to genotype G1. The G1 genotype can develop fertile cysts in sheep, but it can also infect goats, cattle, camel, pigs, and humans. We found only two isolates (3.6%) belonged to genotype G3. This isolates belonged to the cattle samples (2/18, 11.1%). The presence of G3 genotype producing fertile cysts in cattle, suggests the correlation between G3 genotype and producing fertile cysts in cattle and its high host specificity.

There are several studies that showed G1 genotype of *E. granulosus* is the predominant genotype in animals and humans around the world. For example, Utuk et al. (17) conducted a study in Turkey on 208 isolates (179sheep, 19 cattle, seven goats, one camel, one dog, and a single human sample) and detected only the G1 genotype in all of their isolates. Reviews have reported a similar result with different G1 to G3 proportions: for example, 95.5 % G1 vs. 4.5 % G3 in 112 sheep and cattle in Turkey (18); 78.75 % G1 vs. 12.5 % G3 in 80 cattle and water buffaloes in Italy (19); 93.3 % G1 vs. 6.7 % G3 in 30 ovine, bovine, and humans in Tunisia (20); 77.8 % G1 vs. 11.1 % G3 in 18 humans and dogs in southern Brazil (21); and 73.7 % G1 vs. 13.2 % G3 in 38 different intermediate hosts in southeast of Iran (22). Unlikely, Pednekar et al. (23) have demonstrated G3 in 63 % as the predominant genotype, whereas the G1 genotype was found only in six (13 %) isolates from 46 domestic livestock in India.

In line with our findings several investigations have been conducted on genotyping of *E. granulosus* in Iran. For example, dominant strain of *E. granulosus* in Chaharmahal-va –Bakhtyari Province, Central Iran, was G1 (24). Other study conducted in Isfahan Province, central Iran, on hydatid cysts with origination of cattle, sheep, goat, camel and human, showed G1 genotype as the dominant strain (25). Among 86 isolates *E. granulosus* isolated from humans and domestic animals from northwest of Iran (Zanjan Province) using the mitochondrial cox1 gene, 82 (95.35 %) were determined as G1 genotype, and the remaining four (4.65%), were identified as G3 genotype (26). All samples isolated in definitive and intermediate hosts of the *E. granulosus* belonged to G1 genotype (sheep strain) (27). In another study, three distinct genotypes (G1, G3, and G6) were reported from human, sheep, cattle, goats, and camels in Kerman Province, southeast of Iran (22). Ninety three isolated hydatid cysts from slaughtered livestock of Yasuj City, were G1 genotype (28). Moreover, in Ardabil Province, G1 was the dominant strain and for the first time in Iran G3 genotype in 2 human isolates was reported (29).

Based on the previous investigation three genotypes (G1, G2 and G3) were identified from the *E. granulosus* isolates of stray dogs, from Lorestan province using DNA sequencing of the partial mitochondrial *cox*1 and *nad*1 genes, whereas sheep appear to be a principal animal intermediate host for *E. granulosus* in this region. In other studies on human and domestic herbivores of this province, the results showed G1 as the dominant strain indicating the main intermediate host and biological maintenance in the nature are sheep and goat (30, 31). In Lorestan Province, the livestock (sheep and cattle) industry is a major economic component. Once these domestic animals are infected with *E. granulosus*, the habit of feeding raw offal of animals to dogs will easily lead to *E. granulosus* infection in dogs. Thus, this parasite can complete its life cycle in our area.

## Conclusion

The common sheep strain/G1 is predominant genotype in the western part of Iran, followed by G3 genotype, circulating among the animal hosts in this region. The presence of dominant sheep strain (G1) in sheep and cattle of Lorestan Province shows that these animals probably infected of the same origin. This is because of the popularity of traditional live stock in the area that cattle and sheep use from one pasture. Further studies covering a larger number of isolates might be necessary to see if there are other genotypes in the hydatid cyst population in this region of the country.

## References

[1] World Health Organization (WHO) informal working group on echinococcosis. Bull WHO. 1996; 74:231–42. 8789923 PMC2486920

[2] Mahmoudvand H Kheirandish F Dezaki ES Shamsaddini S Harandi MF Chemical composition, efficacy and safety of *Pistacia vera* (var. Fandoghi) to inactivate protoscoleces during hydatid cyst surgery. Biomed Pharmacother. 2016; 82: 393–8. 27470377 10.1016/j.biopha.2016.05.012

[3] Schneider R Gollackner B Schindl M Tucek G Auer H *Echinococcus canadensis* G7 (pig strain): an underestimated cause of cystic echinococcosis in Austria. Am J Trop Med Hyg. 2010; 82(5):871–4. 20439969 10.4269/ajtmh.2010.09-0639 PMC2861383

[4] Thompson RC The taxonomy, phylogeny and transmission of *Echinococcus*. Exp Parasitol. 2008; 119(4):439–46. 18539274 10.1016/j.exppara.2008.04.016

[5] Rostami Nejad M Taghipour N Nochi Z Mojarad EN Mohebbi SR Harandi MF Zali MR Molecular identification of animal isolates of *Echinococcus granulosus* from Iran using four mitochondrial genes. J Helminthol. 2012; 86(4):485–92. 22166311 10.1017/S0022149X1100071X

[6] Sharbatkhori M Mirhendi H Jex AR Pangasa A Campbell BE Kia EB Eshraghian MR Harandi MF Gasser RB Genetic categorization of *Echinococcus granulosus* from humans and herbivorous hosts in Iran using an integrated mutation scanning-phylogenetic approach. Electrophoresis. 2009; 30(15):2648–55. 19637222 10.1002/elps.200900145

[7] Sharbatkhori M Fasihi Harandi M Mirhendi H Hajialilo E Kia EB Sequence analysis of cox1 and nad1 genes in *Echinococcus granulosus* G3 genotype in camels (*Camelus dromedarius*) from central Iran. Parasitol Res. 2011; 108(3):521–7. 20922418 10.1007/s00436-010-2092-7

[8] Rostami Nejad M Nazemalhosseini Mojarad E Fasihi Harandi M Echinococcosis: based on molecular studies in Iran. Gastroenterology Hepatology Bed Bench. 2010; 3: 169–76.

[9] Parsa F Fasihi Harandi M Rostami S Sharbatkhori M Genotyping *Echinococcus granulosus* from dogs from Western Iran. Exp Parasitol. 2012; 132(2):308–12. 22884512 10.1016/j.exppara.2012.07.010

[10] Bowles J Blair D McManus DP Genetic variants within the genus *Echinococcus* identified by mitochondrial DNA sequencing. Mol Biochem Parasitol. 1992; 54(2):165–73. 1435857 10.1016/0166-6851(92)90109-w

[11] Kheirandish F Sharafi AC Kazemi B Bandehpour M Tarahi Mj Khamesipour A First molecular identification of *Leishmania* species in a new endemic area of cutaneous leishmaniasis in Lorestan, Iran. Asian Pac J Trop Med. 2013;6(9):713–7. 23827149 10.1016/S1995-7645(13)60124-8

[12] Kheirandish F Chegeni Sharafi A Kazemi B Mohebali M Sarlak A Tarahi MJ Holakouee K Hajaran H Identification of *Leishmania* species using PCR assay on Giemsa-stained slides prepared from cutaneous leishmaniasis patients. Iran J Parasitol. 2013;8(3):382–8. 24454430 PMC3887238

[13] Kheirandish F Badparva E Haghighi A Nazemalhosseini Mojarad E Kazemi B Differential diagnosis of *Entamoeba* spp. in gastrointestinal disorder patients in Khorramabad, Iran. African J Microbiol Res. 2011; 5(18): 2863–6.

[14] Kheirandish F Tarahi MJ Ezatpour B Prevalence of intestinal parasites among food handlers in West of Iran. Rev Inst Med Trop Sao Paulo. 2014;56(2):111–4. 24626411 10.1590/S0036-46652014000200004 PMC4085852

[15] Badparva E Kheirandish F Ebrahimzade F Prevalence of intestinal parasites in Lorestan Province, West of Iran. Asian Pac J Trop Dis. 2014; 4: 930–4.

[16] Kheirandish F Tarahi M Haghighi A Nazemalhosseini-Mojarad E Kheirandish M Prevalence of intestinal parasites in bakery workers in Khorramabad, Lorestan Iran. Iran J Parasitol. 2011; 6(4): 76–83. 22347316 PMC3279910

[17] Utuk AE Simsek S Koroglu E McManus DP Molecular genetic characterization of different isolates of *Echinococcus granulosus* in east and southeast regions of Turkey. Acta Trop. 2008; 107(2):192–4. 18579101 10.1016/j.actatropica.2008.05.026

[18] Vural G Baca AU Gauci CG Bagci O Gicik Y Lightowlers MW Variability in the *Echinococcus granulosus* cytochrome C oxidase 1 mitochondrial gene sequence from livestock in Turkey and a reappraisal of the G1–3 genotype cluster. Vet Parasitol. 2008; 154(3–4):347–50. 18485601 10.1016/j.vetpar.2008.03.020

[19] Casulli A Manfredi MT La Rosa G *Echinococcus ortleppi* and *E. granulosus* G1, G2 and G3 genotypes in Italian bovines. Vet Parasitol. 2008 8 1;155(1–2):168–72. 18514422 10.1016/j.vetpar.2008.04.004

[20] M’rad S Oudni-M’rad M Filisetti D Molecular identification of *Echinococcus granulosus* in Tunisia: first record of the Buffalo strain (G3) in human and bovine in the country. Open Vet Sci J. 2010; 4:27–30.

[21] de la Rue ML Takano K Brochado JF Costa CV Infection of humans and animals with *Echinococcus granulosus* (G1 and G3 strains) and *E. ortleppi* in Southern Brazil. Vet Parasitol. 2011; 177(1–2):97–103. 21273000 10.1016/j.vetpar.2010.11.018

[22] Hajialilo E Harandi MF Sharbatkhori M Mirhendi H Rostami S Genetic characterization of *Echinococcus granulosus* in camels, cattle and sheep fromthe south-east of Iran indicates the presence of the G3 genotype. J Helminthol. 2012; 86(3):263–70. 21749740 10.1017/S0022149X11000320

[23] Pednekar RP Gatne ML Thompson RC Traub RJ Molecular and morphological characterisation of *Echinococcus* from food producing animals in India. Vet Parasitol. 2009; 165(1–2):58–65. 19632783 10.1016/j.vetpar.2009.06.021

[24] Yoosefi H Hashemzadeh M Aliyari Z Molecular study of hydatid cyst (sheep strain) in Chaahrmohal va Bakhteyari by PCR-RFLP. J Shahrkord Uni. 2007; 9(2):28–33.

[25] Shahnazi M Hejazi H Salehi M Andalib AR Molecular characterization of human and animal *Echinococcus granulosus* isolates in Isfahan, Iran. Acta Trop. 2011; 117(1):47–50. 20858453 10.1016/j.actatropica.2010.09.002

[26] Farhadi M Fazaeli A Haniloo A Genetic characterization of livestock and human hydatid cyst isolates from northwest Iran, using the mitochondrial cox1 gene sequence. Parasitol Res. 2015; 114(12):4363–70. 26280086 10.1007/s00436-015-4673-y

[27] Yakhchali M Mardani K Study on strain variation of *Echinococcus granulosus* in domestic life cycle by amplification of nad-1 gene by PCR-RFLP. Iran J Vet. 2011;7(1):63–8.

[28] Sadri A Moshfe A Doosti A Characterization of isolated hydatid cyst from slaughtered livestock in Yasuj industrial slaughterhouse by PCR-RFLP. J Yasuj Uni. 2012; 17(3):143–52.

[29] Pezeshki A Akhlaghi L Sharbatkhori M Razmjou E Oormazdi H Mohebali M Meamar AR Genotyping of *Echinococcus granulosus* from domestic animals and humans from Ardabil Province, northwest Iran. J Helminthol. 2012;87(4):387–91. 23046636 10.1017/S0022149X1200051X

[30] Parsa F Haghpanah B Pestechian N Salehi M Molecular epidemiology of *Echinococcus granulosus* strains in domestic herbivores of Lorestan, Iran. Jundishapur J Microbiol. 2011; 4(2): 123–30.

[31] Kheirandish F Mahmoudvand H Ahmadinejad M Karimi Rouzbahani A Genetic characterization of human-derived hydatid cysts of *Echinococcus granulosus* in Lorestan Province, Western Iran. Tropical Biomed. 2017; 34(4): 863–9. 33592955

